# Ex vivo optical coherence tomography combined with near infrared targeted fluorescence: towards *in-vivo* esophageal cancer detection

**DOI:** 10.1364/BOE.537828

**Published:** 2024-09-05

**Authors:** Margherita Vaselli, Ruben Y. Gabriels, Iris Schmidt, Andrea J. Sterkenburg, Gursah Kats-Ugurlu, Wouter B. Nagengast, Johannes F. de Boer

**Affiliations:** 1 Department of Physics and Astronomy, LaserLab Amsterdam, Vrije Universiteit de Boelelaan 1081,, Amsterdam, The Netherlands; 2 Department of Gastroenterology and Hepatology, University of Groningen, University Medical Center Groningen, Groningen, The Netherlands; 3 Department of Pathology and Medical Biology, University of Groningen, University Medical Center Groningen, Groningen, The Netherlands

## Abstract

Early detection of (pre)malignant esophageal lesions is critical to improve esophageal cancer morbidity and mortality rates. In patients with advanced esophageal adenocarcinoma (EAC) who undergo neoadjuvant chemoradiation therapy, the efficacy of therapy could be optimized and unnecessary surgery prevented by the reliable assessment of residual tumors after therapy. Optical coherence tomography (OCT) provides structural images at a (sub)-cellular level and has the potential to visualize morphological changes in tissue. However, OCT lacks molecular imaging contrast, a feature that enables the study of biological processes at a cellular level and can enhance esophageal cancer diagnostic accuracy. We combined OCT with near-infrared fluorescence molecular imaging using fluorescently labelled antibodies (immuno-OCT). The main goal of this proof of principle study is to investigate the feasibility of immuno-OCT for esophageal cancer imaging. We aim to assess whether the sensitivity of our immuno-OCT device is sufficient to detect the tracer uptake using an imaging dose (∼100 times smaller than a dose with therapeutic effects) of a targeted fluorescent agent. The feasibility of immuno-OCT was demonstrated *ex-vivo* on dysplastic lesions resected from Barrett’s patients and on esophageal specimens resected from patients with advanced EAC, who were respectively topically and intravenously administrated with the tracer bevacizumab-800CW. The detection sensitivity of our system (0.3 nM) is sufficient to detect increased tracer uptake with micrometer resolution using an imaging dose of labelled antibodies. Moreover, the absence of layered structures that are typical of normal esophageal tissue observed in OCT images of dysplastic/malignant esophageal lesions may further aid their detection. Based on our preliminary results, immuno-OCT could improve the detection of dysplastic esophageal lesions.

## 1. Introduction

The elucidation of molecular pathogenesis of cancers allowed the development of molecular target therapies and immunotherapies and triggered a shift toward personalized medicine [1]. By enabling a transition from the one-size-fits-all to a tailored approach, personalized medicine provided a significant improvement in cancer treatment [2]. The need of predicting treatment outcomes and monitoring treatment success stimulated the advancement of novel diagnostic techniques that enable molecular imaging of key biomolecules and molecularly based events involved with oncogenesis [3].

In particular, the development of targeted fluorescent tracers, which consist of a fluorophore conjugated to a tumor-specific target [4], has opened the doors to fluorescence molecular imaging (FMI) [5]. FMI is an optical imaging technique that uses non-ionizing radiation to provide real-time imaging of specific oncologic targets with a micrometer resolution [6]. A variety of targeted fluorescent tracers have been developed for FMI, and monoclonal antibodies (mAbs) were the first to be translated into patients (immuno-fluorescence) [7]. By targeting tumor cell receptors or signaling proteins, mAbs target specific tumor markers [1].

Several hurdles using fluorescent tracers emitting in the visible-region were identified, including limited penetration depth and conspicuous tissue autofluorescence [8]. To overcome these limitations, fluorophores that emit in the near infra-red (NIR) optical window (700-900 nm), such as IRDye800CW, were developed. Due to reduced scattering and lower tissue autofluorescence in the NIR region, NIR fluorophores ensure increased penetration depth and higher signal-to-noise ratio [9,10]. With NIR fluorescently labelled mAbs, NIR-FMI gained interest for *in-vivo* imaging in numerous applications. Several studies have shown the use of NIR-FMI for identification of tumor tissue in head and neck oncology [11–13], in breast oncology [5,14,15], in gynecological cancer [16], in pulmonary oncology [17] and in gastrointestinal oncology [18–22].

Despite its great potential in detecting (pre)malignant tissue and demarcation of tumor margins, NIR-FMI images lack 3-dimensional morphological structural and architectural information of the tissue. Structural information is particularly valuable to determine depth of tumor invasion, volume within the tissue, and to characterize the tumor microenvironment. Morphological information is available with the use of structural imaging technologies. Optical coherence tomography (OCT) is an imaging technique that provides structural images based on the optical backscatter properties of tissue [23]. Compared to other structural imaging techniques such as ultrasound or computed tomography (CT), OCT achieves a higher resolution and higher soft-tissue contrast, as optical scattering is more varied across soft-tissue than acoustic scattering or x-ray absorption [24]. OCT uses infrared light to provide 3-dimensional imaging with a resolution of ∼10 µm and a penetration depth of 2-3 mm [23].

We hypothesize that the tissue structural context obtained using OCT combined with NIR-FMI information regarding the specific molecular targets in cancer progression, will enhance the sensitivity of identifying tumor tissue, invasiveness, behavior, and growth, and could, ultimately, assist in making clinical decisions in individual patients.

A number of studies previously demonstrated the potential of NIR-FMI for the detection of (pre)malignant esophageal lesions using NIR fluorophores conjugated with peptides [25], or antibodies [20,26]. Additionally, research has shown that OCT can be effectively used for microscopic imaging of the esophageal tract providing detailed visualization of various types of esophageal mucosa [27–35]. Over the past years, efforts have been made to integrate OCT with other imaging techniques for enhanced esophageal imaging such as angle resolved OCT, polarization sensitive elastography, and combined photoacoustic, OCT and ultrasound, respectively [36–38]. However, to date, the combination of OCT with fluorescence imaging in the esophageal tract has been limited to imaging of animal models using fluorophores in the visible spectrum that do not bind to a specific target [39,40].

Here we combined NIR-FMI with OCT (immuno-OCT) and demonstrated its potential for imaging of the human esophagus. For the first time we demonstrated *ex-vivo* immuno-OCT on specimens resected from patients diagnosed with dysplastic Barrett’s esophagus (BE). BE is the only known precursor of esophageal adenocarcinoma (EAC) [41], which is one of the main entities of esophageal cancer, affecting 80% of esophageal cancer patients in the western world [42]. Additionally, we demonstrated *ex-vivo* immuno-OCT on resection specimens of patients with advanced EAC after neoadjuvant chemoradiotherapy (nCRT).

In this study, the antibody bevacizumab conjugated with the NIR cyanine fluorophore IRDye800CW (excitation and emission peaks at 780/805 nm) was used as targeted fluorescent tracer. Bevacizumab targets vascular endothelial growth factor A (VEGFA), a protein expressed in a variety of tumors including EAC stimulating the formation of new blood vessels, which are essential for the supply of nutrients and oxygen to the tumor [43,44]. Bevacizumab-800CW was chosen because of its proven discriminative potential of dysplastic esophageal lesions from non-dysplastic BE tissue [20].

The main goal of this proof of principle study is to investigate the feasibility of immuno-OCT for esophageal cancer imaging. Furthermore, we aim to assess whether the sensitivity of our immuno-OCT device is sufficient to detect the tracer uptake using an imaging dose (∼100 times smaller than a dose with therapeutic effects) of targeted fluorescent agents. Additionally, NIR fluorescence (NIRF) images, obtained with our imaging system, were compared with images from commercially available wide-field fluorescence systems. This feasibility study demonstrates that immuno-OCT allows real-time, co-localized, and simultaneous molecular and structural imaging of the tumor microenvironment in the esophageal tract. This study represents a step toward *in-vivo* use of immuno-OCT for minimally invasive detection of early BE and EAC.

## 2. Methods

### 2.1 Immuno-OCT setup

The immuno-OCT system used in this study has previously been described in detail by our group [45]. In short, this dual-modality system consists of two sub-units (OCT and NIRF imaging unit) and a galvo-based scanner as scanning interface. The OCT system is based on a swept source laser (Axsun Inc., Billerica MA, USA) centered at 1310 nm with a 90 nm bandwidth and 50 kHz A-scan rate. The OCT unit is combined with NIRF excitation light from a single mode diode laser (BrixX 775-50 NB, Omicron-Laserage Laserprodukte GmbH, Rodgau, Germany) via an SMF-28 fiber-based wavelength division multiplexer (WD6513A, Thorlabs Inc., Newton, NJ, USA). The two signals are further guided through a single-mode core of a double-clad fiber via a double-clad fiber coupler (DC1300LEFA Castor Optics, Thorlabs Inc., Newton, NJ, USA) to the sample. OCT light backscattered from the sample is collected through the single-mode core of the double-clad fiber (diameter = 9.0 µm; NA = 0.12). Backscattered NIRF light, which is collected by and travels through the inner cladding of the double-clad fiber (diameter = 105.0 ± 5.0 µm; NA = 0.2), is separated from the inner cladding by the double-clad fiber coupler and detected by a single photon counting module (SPCM-NIR, Excelitas Technologies GmbH, Wiesbaden, Germany). The OCT channel achieves an axial resolution of 12 µm in tissue (n = 1.4). The NIRF channel has a lateral optical resolution of 22.3 µm, determined by the point-spread function of the beam incident on the sample [45].

### 2.2 Study design

We demonstrated *ex-vivo* immuno-OCT in two cohorts of patients, A) diagnosed with dysplastic BE lesions or B) advanced EAC. Institutional review board approval for the study was obtained, and all patients included in the study provided signed informed consent. Immuno-OCT measurements on patients had no consequences for further histopathological analysis. For both groups of patients, fluorescently labeled bevacizumab-800CW was used as tracer.

#### 2.2.1 Cohort A: Barrett’s esophagus endoscopic mucosal resection and endoscopic submucosal dissection specimens

Patients diagnosed with low grade dysplasia, high grade dysplasia or early stage EAC during a diagnostic endoscopic examination were scheduled for either an endoscopic mucosal resection (EMR) or submucosal dissection (ESD) to excise the dysplastic lesion completely. The endoscopically resected esophageal specimens were pinned on a silicone base to prevent tissue shrinkage and make the lumen of the esophagus accessible. Immediately after the endoscopic resection, the excised specimens were sprayed with bevacizumab-800CW (0.1 mg/ml per cm BE). After tracer incubation of five minutes, the specimens were rinsed with sufficient water to remove any tracer excess. Subsequently, all resected specimens were imaged with our immuno-OCT device and with a wide field fluorescence camera (Pearl Trilogy, LI-COR Biosciences Inc., Lincoln, NE, USA) for comparison.

**Fig. 1.**
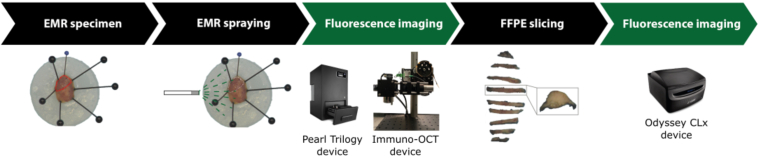
Schematic overview of study design for EMR/ESD specimens. After excision, the resected specimen is pinned to a silicon base, directly sprayed with the tracer and incubated for 5 minutes. After rinsing the specimen with water, fluorescence imaging is performed using the Pearl Trilogy and Immuno-OCT device. After histopathological processing, the 10 µm coupes are imaged with the Odyssey CLx device.

After completing all imaging steps, the specimens were sent to the pathology department of University Medical Centre Groningen (UMCG), sliced into bread loaf slices, and formalin fixated in paraffin-embedded (FFPE) blocks. The FFPE blocks were sliced into 10 µm thick slices, which were imaged with a wide-field fluorescence camera (Odyssey CLx system, LI-COR Biosciences Inc., Lincoln, NE, USA) and afterwards stained with hematoxylin and eosin (HE) and used for histopathological analysis. An overview of the imaging methodology in this study is shown in Figure 1.

#### 2.2.2 Cohort B: advanced esophageal adenocarcinoma specimens

Patients included in this cohort had advanced EAC and underwent nCRT followed by surgical resection of the esophagus (esophagectomy). These patients were part of a dose-finding and feasibility study for the evaluation of preoperative nCRT response using the tracer bevacizumab-800CW (NCT03558724).

All patients received systemic administration of bevacizumab-800CW (25 mg bolus injection) four days before the surgery and imaging procedure. Immediately after the esophagectomy, the resected fresh specimens were cut open along the longitudinal direction of the esophagus by an expert pathologist at the pathology department of the UMCG in order to make the esophageal lumen accessible. The specimens were pinned on a silicone base and then imaged from the luminal side with our immuno-OCT device and with a wide field fluorescence camera (SurgVision Explorer, Bracco Imaging, Milan, Italy). After imaging, the specimens were sent to the pathology department for further macroscopic workup and histopathologic analysis. The tumor stage of the resected specimen (ypTNM) and Mandard score were evaluated.

**Fig. 2.**
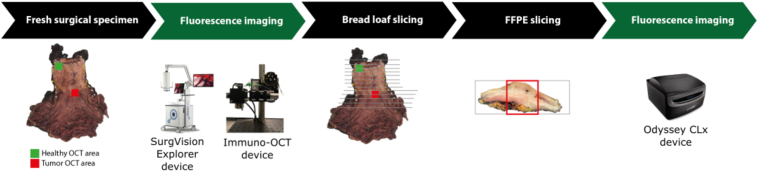
Schematic overview of study design for EAC specimen. Two areas (healthy and tumor area) are selected in the fresh surgical specimen and fluorescence imaging is performed with the SurgVision Explorer and the Immuno-OCT system. Bread loaf slicing and FFPE slicing is performed, and the 10 µm coupes cut from the FFPE blocks are imaged with the Odyssey CLx device.

The specimens were then sliced into bread loaf slices, and (FFPE) tissue blocks were made. From these blocks 10 µm histologic slices were cut, imaged with the Odyssey CLx system (LI-COR Biosciences Inc., Lincoln, NE, USA), HE-stained and used for histopathological analysis. The methodology is illustrated in Fig. 2.

### 2.3 Immuno-OCT data acquisition and image reconstruction

*Ex-vivo* measurements of the resected specimens were performed at the UMCG in a dark room. First, a background measurement for the NIRF was acquired in order to perform background noise subtraction in post-processing. Then, the power of the NIRF and OCT incident lights on the sample were measured before the first scan was acquired. An incident power of 20 mW (775 nm) was used for the NIRF excitation, and an excitation power of 9 mW at the sample(1310 nm, BW 100 nm) was used for OCT*.* For imaging the samples, the galvo-scanner head was positioned above the inner lumen of the esophagus. One-dimensional NIRF and OCT cross sections of the sample were provided by continuously scanning the fast axis galvo mirror and displaying the data in real-time. Real-time imaging of the specimen allowed to position the focus at an optimal depth in the tissue and provided immediate feedback on the quality of the measurement. Once the galvo-scanner head was positioned above a region of interest, a full OCT scan (1000 B-scans of 1000 A-lines) and 2D NIRF images (1000 × 1000 pixels) were simultaneously acquired. Each data acquisition corresponds to a scanned area of 1 × 1 cm^2^, thus the surface dimension of the sample under examination determined the number of scans needed to image it.

Image reconstruction of the raw data was performed in post-processing using MATLAB R2021a (MathWorks). The raw OCT spectra, acquired equally sampled in k-space, were Fourier transformed to create B-scans displayed with a 55 dB dynamic range. To improve the contrast of the OCT images and remove the depth-dependent signal strength, depth-resolved attenuation coefficient (AC) images were produced using an algorithm developed by Vermeer *et al.* [46]. The fluorescence raw data were converted into counts-per-second and a factory-provided correction curve was applied to compensate for the non-linear response of the single photon counting module. NIRF data were displayed in *en-face* images in a color scale, with the lower bound of the color scale being the background and the upper bound the highest count rate among measurements on the same patient group. Moreover, one-dimensional NIRF intensity data were mapped with the co-registered structural (OCT/AC) cross-sectional images and displayed along the scanning axis.

### 2.4 Matching histology with OCT

When scanning the samples, structural (OCT/AC) cross sectional images and NIRF *en-face* images were simultaneously acquired. While structural cross-sectional images are depth-resolved, the co-registered NIRF *en-face* images are not. To understand the source of the NIRF signal, and its depth of origin, structural cross-sectional OCT images revealing the presence of structures within depth were compared with histology and cross sectional NIRF images. A pathologist evaluated the HE-stained histology slides, and the main histological features were highlighted. Next, the structural OCT images of the fresh specimens were visually compared with the HE histology by a researcher with expertise in OCT image interpretation. For each OCT scan acquired, the histologic slide corresponding to the scan location was examined. A match between OCT cross section and HE histology was found when the structural features of one OCT B-scan within one volume scan, corresponded with features in the respective HE histology. The presence of distinctive features (lymph vessels, blood vessels) in some of the histology slides facilitated matching of the corresponding OCT images.

## 3. Results

### 3.1 Dysplastic Barrett’s esophagus study

#### 3.1.1 Study cohort A and imaging procedure

Two patients diagnosed with dysplastic BE tissue were included in the immuno-OCT *ex-vivo* imaging study. Baseline characteristics of these patients are reported in Table 1.

**Table 1.** Patient characteristics of patients diagnosed with early stage of esophageal adenocarcinoma (EAC).

Patient	1	2
Gender	Female	Male
Age	66	82
Resection	ESD	EMR
Histology	EAC	EAC
Nr specimens resected	1	5
Nr of scans per specimen	22	1-4
Specimen size	∼10 × 7 cm	∼1.5 × 1.5 cm

Patient 1 had a submucosal tumor, for which an ESD was performed. In Patient 2 the lesions invasive depth was limited to the mucosal layer of the esophagus and EMR were performed. The number of EMR specimens resected was based on the number of suspicious lesions identified during the endoscopic procedure. All the resected fresh specimens were imaged both with the immuno-OCT system and with a wide field fluorescence camera (Pearl Trilogy). The number of scans performed with the immuno-OCT system on each of the resected specimens varied, depending on the sample dimensions (see Table 1).

#### 3.1.2 NIRF-OCT ex-vivo imaging results

**Fig. 3.**
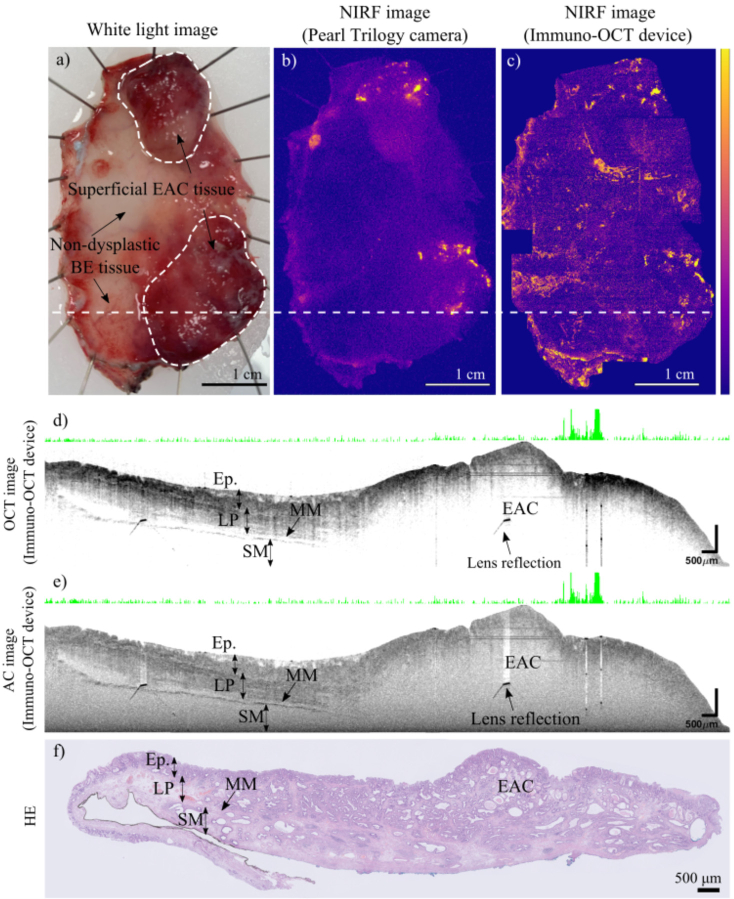
Images of an ESD specimen resected from patient 1, containing superficial EAC tissue and non-dysplastic BE tissue. **a)** White light image of the specimen providing the ground truth validation regarding the lesion site. **b)** NIRF *en-face* image of the specimen acquired with the Pearl Trilogy system **c)** NIRF *en-face* image of the specimen acquired with our immuno-OCT system. The image is a collage of 22 adjacent scans. **d)** OCT cross-section corresponding to the location marked by the white line in Fig. 3(c). The cross-section shows that healthy esophageal tissue (on the left) and EAC (on the right) have different structural properties. The characteristic esophageal layers (Epithelium = Ep., Lamina propria = LP, Muscularis Mucosae = MM and Submucosa = SM) which are preserved in the healthy tissue, are missing in the EAC. **e)** AC cross-section corresponding to the same cross-section of Fig. 3(d). The co-registered 1D NIRF information, is shown in Fig. 3(d-e) as the white band with green peaks whose height is proportional to the intensity of the fluorescence signal. The latter shows a high fluorescence peak corresponding to the EAC margins. **f)** HE-stained slide corresponding to the cross-section in Fig. 3(d-e). Characteristic layers of the esophagus are visible (Ep., LP, MM, SM) on the left, and the EAC is observable on the right.

**Fig. 4.**
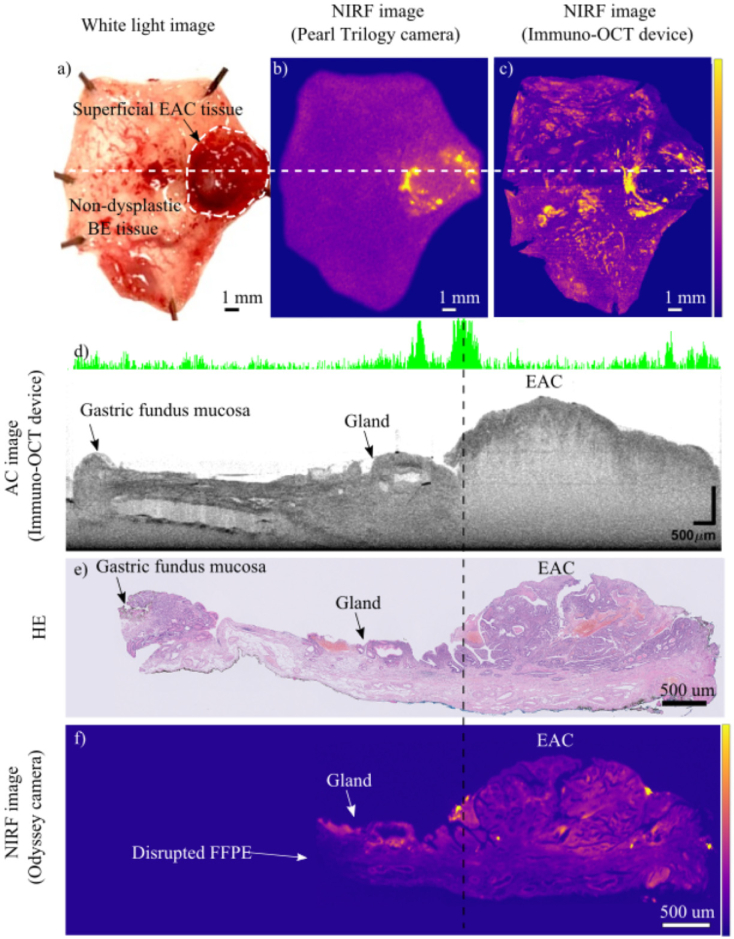
Images of an EMR specimen resected from patient 2, containing superficial EAC tissue and non-dysplastic BE tissue. **a)** White light image of the specimen providing the ground truth validation regarding the lesion site. **b)** NIRF *en-face* image of the specimen acquired with the Pearl Trilogy camera **c)** NIRF *en-face* image of the specimen acquired with our immuno-OCT device. The image is a collage of four adjacent scans. **d)** AC cross-section corresponding to the location marked by the white line in Fig. 4(c). The AC image shows that the scattering properties of the healthy tissue (on the left side of the scan) and those of the tumor (on the right side) are markedly different. In the healthy area of the scan, the layered structure of the EMR specimen and the typical mucosa of the gastric fundus are recognizable. Towards the central part, the specimen presents reactive changes and a gland surrounded by a thin epithelial layer is distinguishable. The right portion of the specimen, characterized by the presence of EAC, shows a loss of the layered structure typical for the esophageal wall. The co-registered 1D NIRF information is shown as the white band with green peaks whose height is proportional to the intensity of the fluorescence signal. **e)** HE-slide corresponding to the AC cross-section in Fig. 4(d). **f)** NIRF *en-face* image acquired with Odyssey camera of the 10 µm slide cut from the same FFPE block of the HE-slide of Fig. 4(f). The left part of the HE coupe is missing since it was damaged during one of the histological preparation steps. It can be noted that the highly fluorescent areas in this image correspond (taking into account tissue shrinking of the HE coupe) to the same axial location where the fluorescence peaks are observed in Fig. 4(d), as pointed out by the dotted line running from Fig. 4(d-f).

Representative images of the ESD specimen (from patient 1) and the EMR specimen (from patient 2) are shown in Figs. 3 and 4 respectively. In both figures, structural images of one cross-section of the specimen are presented. The cross-sections shown were chosen for containing both healthy and dysplastic tissue, and for finding a match with the corresponding HE histology. AC images show superior contrast compared to OCT images as can be seen in Fig. 3. Therefore, AC images are used throughout the rest of the manuscript.

In both ESD and EMR, NIRF *en-face* images which were obtained with both systems consistently show high tracer uptake corresponding to the histologically confirmed malignant or dysplastic lesions (Fig. 3(b-c) and 4(b-c)). NIRF *en-face* images obtained with our system allow to visualize the tracer distribution with superior resolution compared to the ones obtained with the commercial wide field fluorescence camera.

Moreover, our imaging device simultaneously acquired structural (OCT/AC) images, in which morphological differences between healthy and dysplastic/malignant tissue are observed both in ESD and EMR specimens (Fig. 3(d-e) and 4(d-e)).

In OCT cross-sectional images of the normal esophageal wall, the different layers, characteristic for normal esophageal wall tissue, can be distinguished [47–51] (Fig. 3(d)). While OCT images of dysplastic/malignant lesions show a loss of the esophageal wall layered structure [30,52–54] (Fig. 3(d)).

This morphological difference is best appreciated in the depth-resolved AC images (Fig. 3(e) and 4(d)). In AC cross-sections, the use of attenuation coefficient properties of tissue together with the removal of the depth-dependent signal strength, allows to better distinguish the esophageal layers in the healthy tissue. The penetration depth of the structural images is sufficient to visualize all the layers of the esophageal wall of the resected specimens, up to the submucosal layer in case of ESD and up to the mucosa in EMR resections.

When possible, a match between structural images and the 10 µm thick HE histology was found (Fig. 3(f) and 4(e)). In total 12 structural cross-sections (patient 1, n = 7, patient 2, n = 5), could be confidently matched with the corresponding HE histology, enabling histological identification of the structures observed (i.g. lymph vessels, esophageal wall layers, lesions).

The measurements performed with the fluorescent commercial camera (Odyssey CLx) on the 10 µm coupes revealed the cross-sectional distribution of the tracer within the specimen (Fig. 4(f)). Results show that the high fluorescent signal corresponded to the margins of the lesion and were rather superficial due to the topical administration of the tracer.

### 3.2 Advanced esophageal adenocarcinoma study

#### 3.2.1 Study cohort B and imaging procedure

Three patients diagnosed with advanced EAC were included in the *ex-vivo* immuno-OCT imaging sub-study after undergoing nCRT and esophagectomy. Patient characteristics are shown in table 2. All resection specimens included in this study had residual tumor after nCRT, classified according to the TNM stage and using the Mandard tumor regression score.

**Table 2.** Patient characteristics of patients diagnosed with advanced esophageal adenocarcinoma (EAC) including both the clinical TNM stage, pathology TNM stage, and the Mandard tumor regression score after nCRT.

Patient	1	2	3
Gender	Male	Male	Male
Age	65	61	70
Tracer dosage	25 mg	25 mg	25 mg
cTNM stage	cT3N1M0	cT3N0M0	cT3N0M0
ypTNM stage	ypT3N1	ypT3N0	ypT3N0
Mandard	2	3	3
Nr of scans per specimen	15	27	28
Specimen size	∼19 × 5 cm	∼19 × 5 cm	∼19 × 5 cm

All the resected fresh specimens were imaged both with the immuno-OCT system and with a wide field fluorescence camera (SurgVision Explorer). Given the extent of the resected specimens (∼19 × 15 cm) a region of interest for immuno-OCT imaging was selected for each specimen. The region of interest included a non-dysplastic area where no tumor was expected, and a macroscopically suspected tumor area. The number of scans performed with our immuno-OCT device on the resected specimens varied depending on the dimension of the selected region of interest (see table 2).

#### 3.2.2 NIRF and Immuno-OCT ex-vivo imaging results

**Fig. 5.**
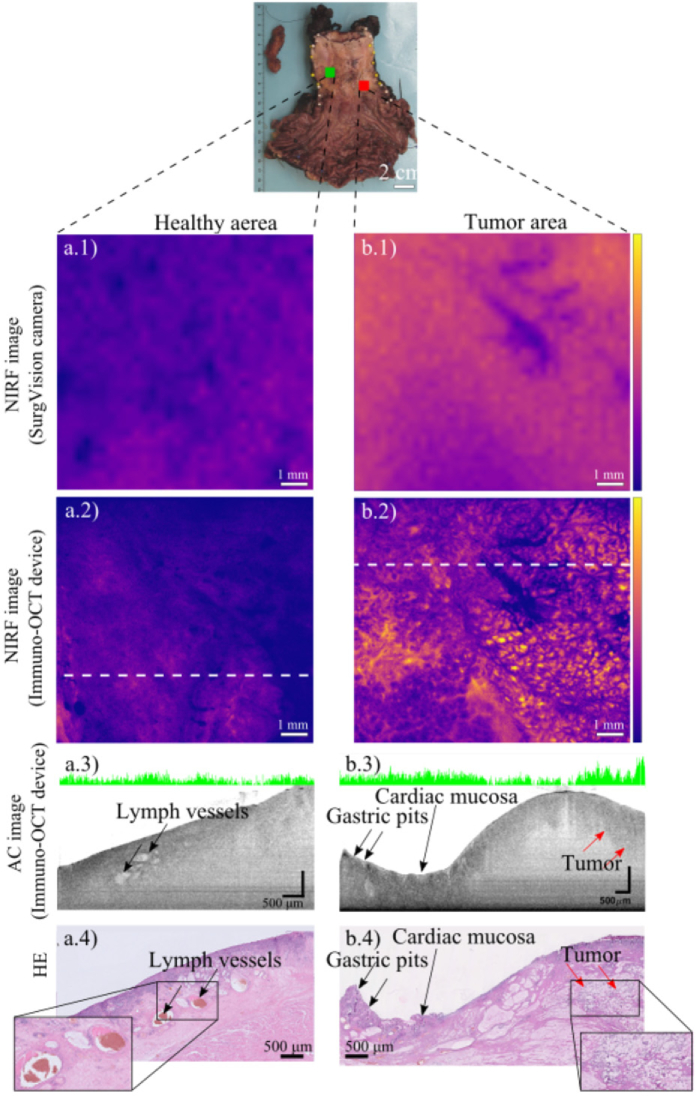
Results of the measurements performed on an EAC specimen resected from patient 3. Images from a healthy area on the esophageal lumen and tumor area in proximity of the gastroesophageal junction of the specimen are shown in Fig. 5(a) and 5(b) respectively. **a.1)** Wide-field NIRF image acquired with SurgVision Explorer camera of a 1 × 1 cm healthy area of the resected specimen. **a.2)** same area scanned with our Immuno-OCT system. **a.3)** AC cross-section corresponding to the location identified by the white line in Fig. 5(a).2. The AC-cross section shows the presence of a few lymph vessels. 1- dimensional NIRF information is displayed by the green lines in the upper part of the image. The length of each green bar is proportional to the NIRF intensity. **a.4)** HE histology corresponding to the AC cross-section in Fig. 5 **b.1)** Wide-field NIRF image acquired with SurgVision Explorer camera of a 1 × 1 cm tumor area of the resected specimen. **b.2)** The same area scanned with our NIRF scanner, shows high tracer uptake in the columnar epithelium of the stomach. **b.3)** AC cross-section corresponding to the location identified by the white line in Fig. 1(b).2. The irregular surface typical of the cardiac mucosa and the presence of gastric pits (low scattering areas within the cardiac mucosa) are visible on the left side of the cross-section. The right side of the cross-section shows the presence of a tumorous area in which no presence of characteristic structure is observed. 1- dimensional NIRF information is displayed by the green lines in the upper part of the image. The length of each green bar is proportional to the NIRF intensity. The one-dimensional NIRF data displayed along the scanning axis indicates high fluorescence signal corresponding to the axial location of the tumor. **b.4)** HE histology corresponding to the AC cross-section in Fig. 1(b).3. Histopathological examination of the HE histology confirmed the presence of tumor cells (indicated by the red arrows).

**Fig. 6.**
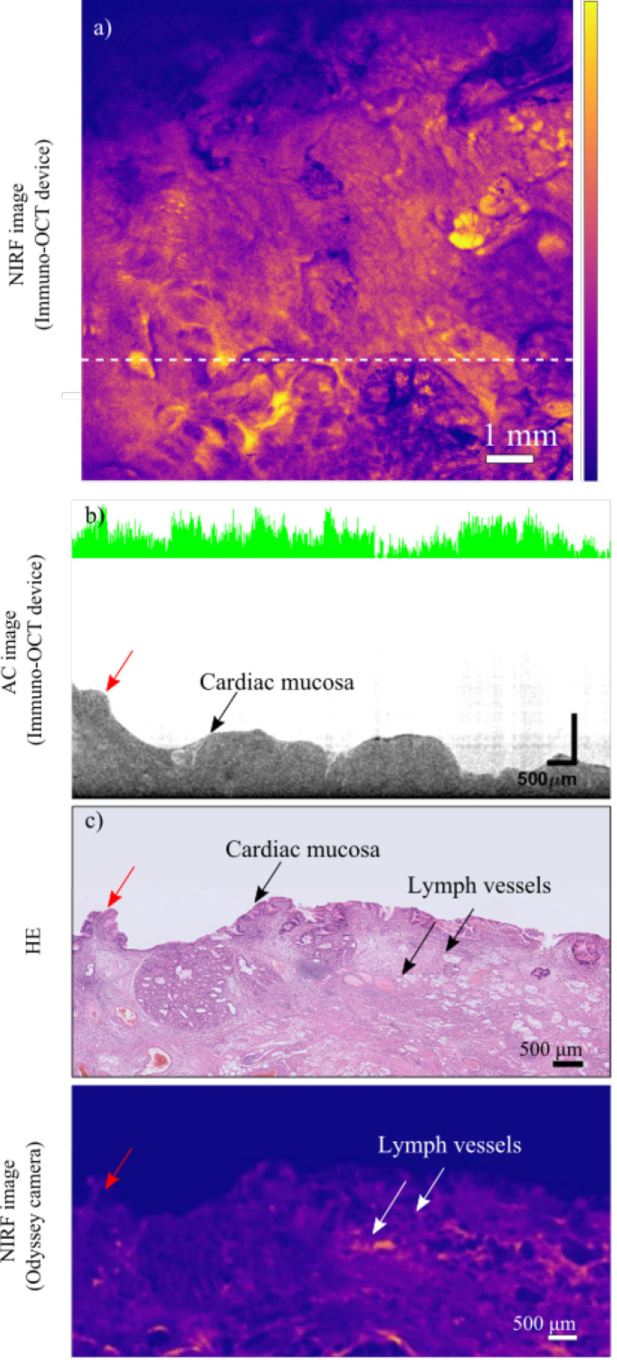
Results of the measurements performed on an inflamed area in proximity of the gastroesophageal junction of the EAC specimen resected from patient 3. **a)** NIRF *en-face* image of the lumen of the resected specimen acquired with our immuno-OCT system **b)** AC cross-section corresponding to the location marked by the white line in Fig. 6(a). Several lymph vessels are identifiable thanks to their lower scattering properties compared to the surrounding tissue, and cardiac type mucosa is distinguishable by its irregular surface. The co-registered 1D NIRF band shows the presence of fluorescence peaks indicating high levels of tracer uptake throughout the tissue. **c)** Histology slide corresponding to the AC cross-section in Fig. 6(b). Histological features visualized in the AC cross-sectional image of Fig. 6(b) are observed. The elevated number of lymph vessels and the amount of mucus suggests that the tissue is extremely reactive and inflamed. **d)** NIRF *en-face* image acquired with the Odyssey camera of the 10 µm slice cut from the same FFPE block from which the HE slide of Fig. 6(c) was cut. This enables visualization of the tracer distribution within the tissue and shows the presence of high fluorescence in correspondence with inflamed tissue, which presence is confirmed by the high amount of lymph vessels.

Images of the resection specimen of patient 3 have been chosen as representative imaging results of the *ex-vivo* study on the EAC patients (Fig. 5 and 6).

In Figure 5, images from a healthy area in the esophageal mucosa (Fig. 5(a)) and from a tumor area in the proximity of the gastroesophageal junction (Fig. 5(b)) are compared. Figure 6 shows the results of the measurements performed on an inflamed area of the specimen.

In both figures, structural (AC) images of one cross-section of the specimen are presented (Fig. 5(a.3, b.3) and Fig. 6(b)). The cross-sections shown were chosen for being among the structural cross-sectional images for which a match with the corresponding HE histology was found.

All resected specimens were imaged with the fluorescent wide field camera and with our immuno-OCT imaging system (Fig. 5(a.1-2, b.1-2) and Fig. 6(a)). NIRF *en-face* images obtained with both systems are consistent with each other. NIRF *en-face* images show high tracer uptake in the tumor bed, where the residual tumor is located (Fig. 5(b.1-2)). Tissue areas histologically identified as non-dysplastic BE tissue show low fluorescence signal (Fig. 5(a.1-2)). However, high fluorescence signals are also observed in NIRF *en-face* images of certain areas of the tumor bed without rest tumor but with highly reactive and inflamed tissue in histological examination (Fig. 6(a)).

NIRF *en-face* images obtained with our system allow to visualize the tracer distribution with superior resolution compared to the ones obtained with the commercial camera. Especially in images of the tumors and of inflamed areas, our imaging system revealed the tracer distribution to be extremely heterogeneous. On the contrary, in healthy tissue, the tracer distribution appeared rather uniform.

Structural images simultaneously acquired with the immuno-OCT system, show morphological differences between healthy and tumor tissues (Fig. 5(a.3, b.3) and Fig. 6(b)). Structural images of healthy and inflamed areas of the resected specimens, showed the presence of gastric mucosa, visualized the gastric pits and lymph and blood vessels [47,50,55], while tumor areas were recognizable in structural images as protruding areas with rather homogeneous scattering properties [30,52,53,56].

When possible, a match between structural images and the 10 µm thick HE histology was found (Fig. 5(a.4, b.4) and Fig. 6(c)). In total 16 structural cross-sections (patient 1, n = 4, patient 2, n = 4, patient 3, n = 8), could be convincingly matched with the corresponding HE histology, enabling histological identification of the structures observed (i.g. lymph vessels, esophageal/gastric mucosa, tumor areas).

Measurements performed with the fluorescent commercial flatbed scanner (Odyssey) on the 10 µm coupes revealed high tracer uptake within the tissue in proximity of tumor and inflamed tissues (Fig. 6(b)). In these cases, given the systemic administration of the tracer, high-fluorescent signal was observed, not only superficial but also within the tissue depth.

## 4. Discussion

To the best of our knowledge, this is the first demonstration of immuno-OCT, the combination of OCT with NIR-FMI, for imaging of human esophageal tissue. While OCT and NIR-FMI can individually provide clinically valuable information, we showed that the combination of these modalities could potentially enhance visualization of (pre)malignant esophageal lesions and improve evaluation of therapy response in individual patients.

Architectural tissue information provided by OCT is of critical importance to distinguish tissue types and evaluate tumor infiltration in the mucosa and submucosa. Consistent with previous studies, our results indicate that OCT cross-sectional images of healthy esophageal tissue preserve the typical layered structure of the esophageal wall, allowing for the distinction of different layers [32,47–49,33]. OCT cross-sections of the gastric cardia visualized the gastric mucosa, whose surface is characterized by the presence of gastric pits [27,47]. In line with earlier research [30,34,52,53], the layered structure typical of healthy esophageal tissue is absent in OCT images of dysplastic and malignant lesions, where the tissue appears more homogenous. This morphological distinction between healthy and dysplastic esophageal tissue is even more clear in AC images which show the scattering properties of tissue.

NIR-FMI with the targeted tracer bevacizumab-800CW provides tissue molecular information and has potential to improve the detection of dysplastic BE and EAC lesions. In a previous study, the NIR-FMI detection limit of our immuno-OCT system was assessed by measuring solutions with increasing concentration of IRDye800CW [45]. The detection sensitivity found (0.3 nM) was hypothesized to be sufficient to detect antibody concentrations anticipated in tumors in humans injected with a micro-dose of immuno-imaging tracers. In the current study, we have shown that the NIR-FMI detection sensitivity of our immuno-OCT system allows to visualize the tracer uptake by topical administration or by injection of an imaging dose of targeted fluorescent tracer [45].

Moreover, NIRF images acquired with our immuno-OCT system were visually compared with NIRF images acquired with commercially available fluorescence cameras. Commercially available cameras offer the advantage of providing wide field NIRF images, which enable to image the entire sample at once, without the need of scanning through it. However, NIRF images acquired with our immuno-OCT system provided higher-resolution (∼10 µm) molecular information and revealed with higher detail compared to commercially available fluorescence cameras the heterogeneity of the tracer distribution.

In BE patients, high NIR-FMI signal was observed at the margins of lesions histologically confirmed as dysplastic. The accumulation of the tracer may indicate a higher presence of VEGFA in the microenvironment of the lesions.

In patients with advanced EAC, the use of systemic administration of bevacizumab-800CW (homogenously distributed within the tissue) enhanced the differences in NIR-FMI signal between the benign mucosal lining and high-grade dysplasia-EAC areas. Benign tissue showed low tracer uptake. In high-grade dysplasia-EAC areas, a high and heterogeneous tracer uptake was observed. However, EAC patients who received systemic administration of the tracer showed high NIR-FMI signal in areas of inflammation. Tissue inflammation is a consequence of the radiation therapy that could affect bevacizumab-800CW biodistribution within the tissue. Ongoing studies are investigating the overexpression of VEGF in inflamed tissue with revascularization, which might explain the accumulation of bevacizumab-800CW even in absence of tumor tissue.

In this study, the potential of immuno-OCT imaging was demonstrated using two different forms of tracer administration. In BE patients the EMR/ESD specimen was sprayed with the tracer *ex-vivo.* In patients with advanced EAC, treated with nCRT and scheduled for esophagectomy, the tracer was intravenously administered prior to surgery. Although we performed *ex-vivo* spraying of the tracer on EMR/ESD specimens, this method could be translated to *in-vivo*, in human, during an endoscopic procedure by topical administration using a spray catheter that goes through the working channel of the endoscope [20]. Topical administration of the tracer would be preferred for diagnostic purposes since it does not require waiting a few days before the imaging procedure and does not impact the standard gastroenterology workflow. Previous studies have shown that topical administration of bevacizumab-800CW can be used as a red flag imaging technique to improve detection of flat and invisible esophageal lesions (19). On the other hand, intravenous agent administration infiltrates deeper in sub-surface tissues that are not accessible by topical administration. Therefore, systemic administration could be used to gain insight into drug distribution of targeted agents for esophageal cancer treatment [57].

Although the limited number of patients is a limitation of this study, we believe this work serves as valuable proof of principle study. Results were validated with 1 to 1 matches of structural cross sections with corresponding histology, which confirmed the presence or absence of dysplastic BE/EAC.

We envision this work as a preliminary step toward endoscopic use of immuno-OCT *in-vivo* use in humans. *In-vivo* NIR-FMI endoscopic imaging following topical or systemic administration of bevacizumab-800CW has been shown to enhance dysplastic and EAC lesions detection rates compared to conventional white light endoscopy [20]. Furthermore, unlike other endoscopic multi-modal OCT-based technologies, such as OCT combined with autofluorescence imaging (AFI) [58], immuno-OCT benefits from the molecular specificity of NIR-FMI and is not hindered by background fluorescence from normal tissues.

In the past decade, the development of OCT tethered capsules emerged as a promising minimally invasive method for three-dimensional microscopic imaging of the esophagus [33,59–63]. Merging NIR-FMI imaging with OCT in tethered capsules would enable visualization of surface patterns of the tracer distribution in the esophagus while simultaneously providing cross-sectional structural images.

Immuno-OCT endoscopy might further enhance the diagnosis of dysplastic BE/EAC by contextualizing the molecular information within the tissue anatomic structure. However several challenges should be addressed for its clinical adoption. The integration of OCT with NIR-FMI requires sophisticated equipment and expertise, which can increase the complexity and cost of the procedure. Furthermore, the effectiveness of immuno-OCT depends on the availability and specificity of fluorophores that can target relevant biomarkers, which can limit its applicability if suitable fluorophores are not available.

## 5. Conclusion

In this feasibility study, we have shown that immuno-OCT has the sensitivity to detect the fluorescence signals with high resolution of fluorescently labelled antibodies administered topically or intravenously. Furthermore, we demonstrated the potential of immuno-OCT to improve esophageal lesion detection by combining the visualization of *en-face* molecular tracer uptake with structural cross-sectional tissue imaging of the esophagus *ex-vivo*. This represents the first step toward endoscopic immuno-OCT for *in-vivo* imaging of human esophagus. The latter, upon further validation, could potentially enable minimally invasive detection of early esophageal dysplastic lesions and evaluate nCRT therapy effectiveness in patients with advanced EAC.

## Data Availability

Data underlying the results presented in this paper are not publicly available at this time but may be obtained from the authors upon reasonable request.
